# Examining the Correlation between Emotional Intelligence and Burnout: A Study on Mental Health in the Workplace

**DOI:** 10.1192/j.eurpsy.2025.2163

**Published:** 2025-08-26

**Authors:** R. Galea, A. Zahra, E. Cassar, K. J. Farrugia, C. Busuttil, B. Gatt, R. Micallef, M. Micallef

**Affiliations:** 1 Mount Carmel Hospital, Mental Health Services; 2 Mater Dei Hospital, Health Services, Attard, Malta

## Abstract

**Introduction:**

Mental Health (MH) in the workplace profoundly influences employee well-being. Burnout, Emotional Exhaustion (EE), depersonalization and diminished personal accomplishment are prevaling concerns among healthcare professionals. Left unchecked these facets can impinge on professionals’ MH and productivity.

**Objectives:**

This study explores the relationship between Emotional Intelligence (EI) and burnout among staff at Mount Carmel Hospital by assessing the degree of EE, depersonalisation and personal accomplishment before and after the EI training.

**Methods:**

121 participants from four hospital wards were recruited. An anonymized questionnaire assessed EI, burnout and demographics including the Schutte Self Report EI Test and the Maslach Burnout Inventory. EI training was provided by professionals from within the hospital service. Post-training measures were reassessed to determine the training’s impact on improving the constructs of occupational burnout.

**Results:**

Gender analysis revealed higher EI scores among females (125.4±11.2) compared to males (117.0±13.9, p=0.026). EE was significantly higher among Maltese staff (20.4±8.3) compared to EU (17.2±4.8) and third-country nationals (12.3±4.9, p=0.027). Longer ward tenure (11-25 years) correlated with higher EE (32.7±6.8) compared to <1 year (16.6±8.1) or 1-5 years (17.6±5.7, p=0.0087). Negative correlations between EI and depersonalization (r=-0.32, p<0.01) emerged, indicating higher EI is associated with lower levels of depersonalization. A significant positive correlation between EI and personal achievement (r=0.54, p<0.01) suggested that higher EI is associated with higher levels of personal achievement. No correlation was found between EI and EE. Phase two revealed a significant difference in post-training EI. Other measures showed no significance; suggesting that other corporate fundational aspects impinge on employee MH.

The findings have important implications for MH professionals and organisations. The correlation between EI and burnout highlights the importance of promoting EI. Specific correlations need further testing, as the higher degree of EE in participants with higher scores of EI effects training development. Training programs focusing on EI should be incorporated into continous professional development. Significant variations in EE were observed among participants from different nationalities and those with varying years of experience in the ward; underscoring the importance of tailored interventions enhancing EI and mitigating burnout.

**Conclusions:**

This study provides evidence for the correlation between EI and constructs of burnout among staff at Mount Carmel Hospital. Further corporational functional aspects potentially impact employees’ professionals outlook, perception and MH. Training programs aimed at enhancing EI can be used to reduce and mitigate burnout levels while improving well-being in the workplace.

**Disclosure of Interest:**

None Declared

